# Concurrence of Inactivation Enzyme-Encoding Genes *tet*(X), *bla*_EBR_, and *estT* in *Empedobacter* Species from Chickens and Surrounding Environments

**DOI:** 10.3390/foods13193201

**Published:** 2024-10-09

**Authors:** Chong Chen, Yilin Lv, Taotao Wu, Jing Liu, Yanan Guo, Jinlin Huang

**Affiliations:** 1 Joint International Research Laboratory of Agriculture and Agri-Product Safety, Ministry of Education of China, Institutes of Agricultural Science and Technology Development, Yangzhou University, Yangzhou 225009, China; chen_chong@yzu.edu.cn (C.C.); 15050731151@163.com (Y.L.); wtt1493270230@163.com (T.W.); 17851971707@163.com (J.L.); 2 Jiangsu Key Laboratory of Zoonosis, Jiangsu Co-Innovation Center for Prevention and Control of Important Animal Infectious Diseases and Zoonoses, Yangzhou University, Yangzhou 225009, China; 3 Key Laboratory of Prevention and Control of Biological Hazard Factors (Animal Origin) for Agrifood Safety and Quality, Ministry of Agriculture of China, Yangzhou University, Yangzhou 225009, China; 4 Animal Science Institute, Ningxia Academy of Agriculture and Forestry Sciences, Yinchuan 750002, China; gyn330@126.com

**Keywords:** *Empedobacter* sp., chicken, tetracyclines, *tet*(X), β-lactams, *bla*_EBR_, macrolides, *estT*, IS*CR2*

## Abstract

The emergence of inactivation enzyme-encoding genes *tet*(X), *bla*_EBR_, and *estT* challenges the effectiveness of tetracyclines, β-lactams, and macrolides. This study aims to explore the concurrence and polymorphism of their variants in *Empedobacter* sp. strains from food-producing animals and surrounding environments. A total of eight *tet*(X) variants, seven *bla*_EBR_ variants, and seven *estT* variants were detected in *tet*(X)-positive *Empedobacter* sp. strains (6.7%) from chickens, sewage, and soil, including 31 *Empedobacter stercoris* and 6 novel species of Taxon 1. All of them were resistant to tigecycline, tetracycline, colistin, and ciprofloxacin, and 16.2% were resistant to meropenem, florfenicol, and cefotaxime. The MIC_90_ of tylosin, tilmicosin, and tildipirosin was 128 mg/L, 16 mg/L, and 8 mg/L, respectively. Cloning expression confirmed that *tet*(X6) and the novel variants *tet*(X23), *tet*(X24), *tet*(X25), *tet*(X26), and *tet*(X26.2) conferred high-level tigecycline resistance, while all of the others exhibited relatively low-level activities or were inactivated. The bacterial relationship was diverse, but the genetic environments of *tet*(X) and *bla*_EBR_ were more conserved than *estT*. An IS*CR2*-mediated *tet*(X6) transposition structure, homologous to those of *Acinetobacter* sp., *Proteus* sp., and *Providencia* sp., was also identified in Taxon 1. Therefore, the *tet*(X)-positive *Empedobacter* sp. strains may be ignored and pose a serious threat to food safety and public health.

## 1. Introduction

With the global spread of carbapenem resistance and colistin resistance, tigecycline has become one of the last-resorts for treating multidrug-resistant (MDR) bacterial infections [1]. Since approval by the United States Food and Drug Administration (USFDA) in 2005, tigecycline has been applied to treating acute bacterial skin and skin structure infections, complicated intra-abdominal infections, and community-acquired bacterial pneumonia worldwide [2]. On the other hand, a flavin-dependent monooxygenase Tet(X) can modify tigecycline through hydroxylation with NADPH, Mg^2+^, and molecular oxygen, leading to its low-level degradation [3]. Recently, the rapid emergence of high-level tigecycline resistance genes *tet*(X3) [4], *tet*(X4) [5], *tet*(X5) [6], *tet*(X6) [6,7], *tet*(X7) [8], and *tet*(X15) [9] in food-producing animals has compromised the clinical efficacy of tigecycline, especially in China. At present, the novel *tet*(X) variants and their transmission risks urgently need to be further evaluated.

The *Weeksellaceae* family is a complex group of Gram-negative, non-spore-forming, non-flagellated, aerobic, microaerobic, or facultatively anaerobic bacteria. It comprises the genera *Empedobacter*, *Weeksella*, *Riemerella*, *Chryseobacterium*, and *Elizabethkingia*, which formerly belonged to the *Flavobacteriaceae* family acting as the ancestral source of *tet*(X) genes [10,11,12]. Despite the few reports of *Empedobacter* sp. strains, *Empedobacter stercoris* was mainly recovered from animal fecal samples, while *Empedobacter falsenii* has become an opportunistic pathogen implicated in clinical blood, ear discharge, pleural fluid, pus, wound, vagina, respiratory tract, urinary tract, and stool samples [13,14,15,16,17,18]. Worrisomely, *Empedobacter* sp. strains exhibit resistance to multiple clinically important antibiotics, such as colistin, carbapenems, and tigecycline [16,17,18]. To the best of our knowledge, these strains are naturally resistant to colistin and carried a metallo-β-lactamase gene *bla*_EBR_ [16,18,19,20]. The decreased tigecycline susceptibility mediated by *tet*(X2), *tet*(X3.2), *tet*(X14), and one unnamed *tet*(X) variant has also been sporadically detected in *E. stercoris*, *E. falsenii*, and *Empedobacter brevis* isolates from human, cattle, pig, shrimp, and chicken samples [10,17,21,22,23]. In 2023, an α/β-hydrolase EstT that inactivates 16-atom-containing macrolides was first reported in *Sphingobacterium faecium* [24]. However, the molecular polymorphism of inactivation enzyme-encoding genes *tet*(X), *bla*_EBR_, and *estT* in MDR *Empedabacter* sp. strains remains poorly understood.

Herein, we intend to explore the concurrence of multiple *tet*(X), *bla*_EBR_, and *estT* variants in *Empedobacter* sp. strains from chickens, sheep, sewage, and soil in Shandong, Jiangsu, and Ningxia provinces in China, followed by analyses of bacterial antimicrobial susceptibility, phylogenetic relationship, genetic diversity, clonal expression level, and antibiotic resistance gene transferability.

## 2. Materials and Methods

### 2.1. Bacterial Isolation and Identification

A total of 556 non-duplicate samples were collected from three chicken farms, one sheep farm, one live poultry market, and one chicken slaughterhouse between 2021 and 2023 in Shandong, Jiangsu, and Ningxia provinces of China (Table S1). These included fecal swabs of chickens (*n* = 317) and sheep (*n* = 95), carcass swabs of chickens (*n* = 60), and samples from the surrounding environment (soil, *n* = 48; sewage, *n* = 36). After dilution with 0.9% physiological saline at a weight/volume or volume/volume ratio of 1/5, 100 μL of them was spread evenly on Luria–Bertani agar (LBA, HuanKai, Guangzhou, China) containing tigecycline (2 mg/L). The *tet*(X)-positive strains were isolated via PCR detection of *tet*(X), and 16S rDNA sequencing was used for bacterial identification (Table S2).

### 2.2. Antimicrobial Susceptibility Testing

According to the Clinical and Laboratory Standards Institute (CLSI) guideline for *Enterobacterales* [25], minimum inhibitory concentrations (MICs) of 12 antibiotics against the *tet*(X)-positive *Empedobacter* sp. isolates were determined via two-fold agar dilution, including tetracycline (dilution range, 0.125–256 mg/L), amikacin (0.5–256 mg/L), gentamicin (0.25–256 mg/L), trimethoprim-sulfamethoxazole (10–160 mg/L), ciprofloxacin (0.004–64 mg/L), colistin (0.25–256 mg/L), cefotaxime (0.125–256 mg/L), meropenem (0.03125–64 mg/L), florfenicol (2–256 mg/L), tylosin (0.25–512 mg/L), tilmicosin (0.125–256 mg/L), and tildipirosin (0.125–256 mg/L). Additionally, the MIC of tigecycline (0.03125–64 mg/L) was determined via broth dilution and interpreted using the USFDA’s criteria for *Enterobacterales* [26]. *Escherichia coli* ATCC 25,922 was used as the quality control.

### 2.3. Whole-Genome Sequencing (WGS)

The genomic DNA of 37 *tet*(X)-positive *Empedobacter* sp. isolates was extracted using a TIANamp Bacteria DNA Kit (Tiangen, Beijing, China) and sequenced using an Illumina NovaSeq 6000 (ANOROAD, Beijing, China). The raw data were assembled using SPAdes version 3.12.0 and assessed using CheckM version 1.1.6 [27,28]. To obtain the complete sequences, *E. stercoris* YWS9-3 [*tet*(X2)- and *tet*(X26)-positive] was further subjected to Oxford Nanopore PromethION (BENAGEN, Wuhan, China), followed by assembling with Unicycler version 0.5.0 and correcting with Pilon version 1.12 [29,30]. The whole-genome sequences of *tet*(X)-positive *Empedobacter* sp. strains deposited in the National Center for Biotechnology Information (NCBI) database were also collected [31].

### 2.4. Bioinformatics Analyses

The Average Nucleotide Identity (ANI, >95%)-based bacterial species and Single Nucleotide Polymorphism (SNP)-based phylogenetic tree of *tet*(X)-positive *Empedobacter* sp. strains were analyzed using Integrated Prokaryotes Genome and pan-genome Analysis (IPGA) version 1.09 [32,33]. Antibiotic resistance genes (ARGs) were analyzed using ResFinder version 4.5.0, with a cutoff at 80% nucleotide identity and 60% nucleotide coverage, and a heatmap was generated with the phylogenetic tree using ggtreeExtra version 1.14.0 [34,35]. The allele numbers of the novel *tet*(X), *bla*_EBR_, and *estT* variants were assigned in the same manner as previously reported [36]. Multiple sequence alignment of Tet(X), EBR, and EstT variants was performed using ESPript version 3.0, respectively [37], with the secondary structure elements of Tet(X2) (Protein Data Bank accession number: 2XDO), EBR-4 (GenBank accession number: MN997121), and EstT-1 (GenBank accession number: CP094932) as their templates. Homology modeling of Tet(X) proteins was based on the published Tet(X2)-tigecycline complex (4A6N) with the online server SWISS-MODEL using default parameters [38]. Accurate structures of EBR and EstT proteins were not available, and therefore, they were predicted using the powerful server AlphaFold 3 [39]. A maximum likelihood tree of Tet(X) variants was constructed with 100 bootstraps using MEGA-X version 10.1.8 and visualized using FigTree version 1.4.4 [40]. Genome sequences of all *tet*(X)-positive *Empedobacter* sp. isolates were annotated using RAST version 2.0, and the genetic environments of *tet*(X), *bla*_EBR_, and *estT* genes were generated with Easyfig version 2.2.5 [41,42].

### 2.5. Cloning Expression

The novel *tet*(X), *bla*_EBR_, and *estT* variants were ligated with an L-arabinose-induced plasmid pBAD24 vis homologous recombination and then transformed into *E. coli* JM109 via heat shock as previously reported [11]. Except for the *Nhe* I- and *Sal* I-digested pBAD24 for *estT-1.3*, *estT-1.4*, *estT-2*, *estT-3*, *estT-4*, and *estT-5* genes, all of the others were treated with *EcoR* I and *Sal* I. The putative transformants were selected on LBA containing ampicillin (100 mg/L) and confirmed using PCR and Sanger sequencing [43]. The amplification primers are listed in Table S2.

Thereafter, the MICs of tetracycline, tigecycline, doxycycline (0.125–256 mg/L), and minocycline (0.125–256 mg/L) against the novel *tet*(X) clones were determined via broth microdilution according to CLSI guidelines [25], with an addition of 0.1% L-arabinose. Our previously reported *tet*(X2), *tet*(X6), and empty clones were used as the control groups [44]. In addition, the MICs of meropenem and cefotaxime against the novel *bla*_EBR_ clones and the reference *bla*_EBR-4_ clone were determined. The MICs of the 16-atom-containing tylosin, tilmicosin, and tildipirosin against the *estT* clones were also determined.

### 2.6. Conjugation Experiments

The transferability of *tet*(X)-mediated tigecycline resistance from *Empedobacter* sp. was determined via filter mating with the recipient *Acinetobacter baylyi* ADP1 (rifampicin-resistant) and *E. coli* C600 (streptomycin-resistant). The putative transconjugants were selected on LBA containing tigecycline (2 mg/L) supplemented with rifampin (100 mg/L) or streptomycin (1500 mg/L). Thereafter, all of them were further screened for *tet*(X) genes and confirmed using PCR fingerprints for *A. baylyi* and enterobacterial repetitive intergenic consensus PCR (ERIC-PCR) for *E. coli* [45,46]. Transfer efficiencies were calculated based on the colony counts of the transconjugant and recipient cells in triplicate [47]. However, the conjugation experiments of *bla*_EBR_ and *estT* genes were not conducted because of their weak inactivation activities.

## 3. Results

### 3.1. Sporadic Distribution of tet(X)-Positive MDR Empedobacter sp. Isolates

Among the 556 samples, a total of 37 *tet*(X)-positive *Empedobacter* sp. strains (6.7%) were isolated from chicken feces (7.9%, 25/317), sewage (30.6%, 11/36), and soil (2.1%, 1/48), but they were negative in chicken carcasses obtained after slaughter and sheep feces (Table S1). These strains consisted of 31 *E. stercoris* and 6 novel species Taxon 1, and the latter shared <95% ANI values with the reference *Empedobacter* species including *E. falsenii* 1681-1 (GCA_013488205.1), *E. stercoris* ES183 (GCA_014621655.1), *E. brevis* ATCC 43319 (GCA_000382425.1), *Empedobacter tilapiae* MRS2 (GCA_004785645.1), and *Empedobacter sedimenti* DT-LB-19 (GCA_030005555.1). By geography, they were mainly *distributed* in Shandong (11.7%, 34/291) but with few in Jiangsu (1.8%, 3/170) and Ningxia (0.0%, 0/95). The results of antimicrobial susceptibility testing showed that all *tet*(X)-positive *E. stercoris* and Taxon 1 strains were MDR. To be specific, 100% of them were resistant to tigecycline, tetracycline, colistin, and ciprofloxacin but remained susceptible to gentamicin, amikacin, and trimethoprim/sulfamethoxazole (Figure S1). Subsets were also resistant to meropenem (16.2%, 6/37), florfenicol (16.2%, 6/37), and cefotaxime (16.2%, 6/37). Meanwhile, the MIC_90_ of tylosin, tilmicosin, and tildipirosin was 128 mg/L, 16 mg/L, and 8 mg/L, respectively (Figure S2).

**Figure 1:**
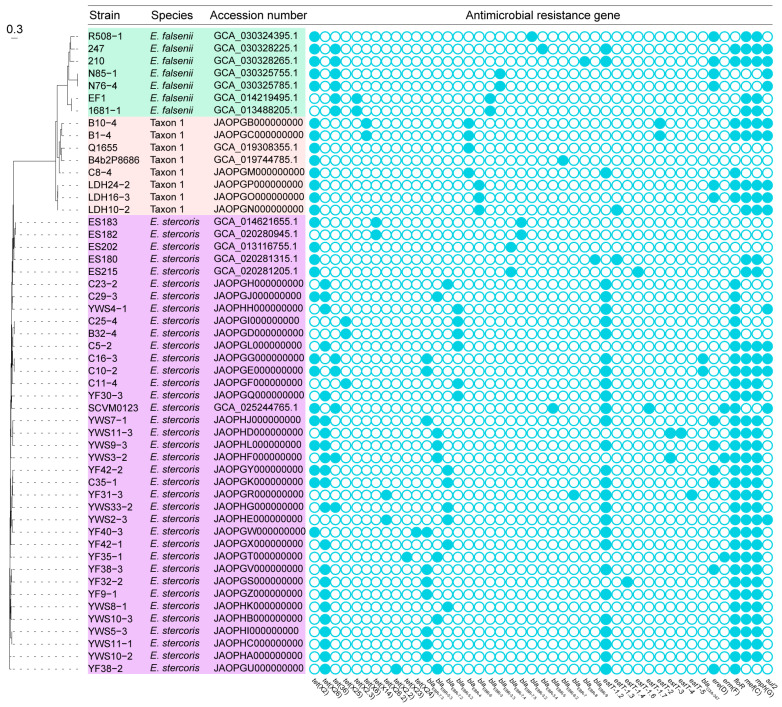
Phylogenetic relationship and heatmap of *tet*(X)-positive *Empedobacter* sp. isolates. The bacterial strains belonging to *E. falsenii*, *E. stercoris*, and Taxon 1 are marked in green, purple, and pink, respectively. Their accession numbers and antibiotic resistance genes (solid circles) are also present. Bar, 0.3 nucleotide substitutions per site.

Based on the WGS data of 37 *tet*(X)-positive *Empedobacteri* sp. strains, in silico mining showed that they carried five classes of ARGs for tetracyclines [*tet*(X), *n* = 46; *tet*(36), *n* = 4], β-lactams (*bla*_EBR_, *n* = 37; *bla*_XA-347_, *n* = 2), macrolides [*estT*, *n* = 34; *mef*(C), *n* = 30; *mph*(G), *n* = 30; *ere*(D), *n* = 8; *erm*(F), *n* = 2], phenicols (*floR*, *n* = 36), and sulfonamides (*sul2*, *n* = 10) (Figure 1). For concurrence events, 24.3% of *tet*(X)-positive *Empedobacter* sp. isolates carried two *tet*(X) variants (e.g., B1-4) and 5.4% of them carried two *estT* variants (e.g., YWS11-3), while none of them carried more than two variants (Figure 1).

### 3.2. Polymorphism of tet(X), bla_EBR_, and estT Variants in Empedobacter Species

**Figure 2:**
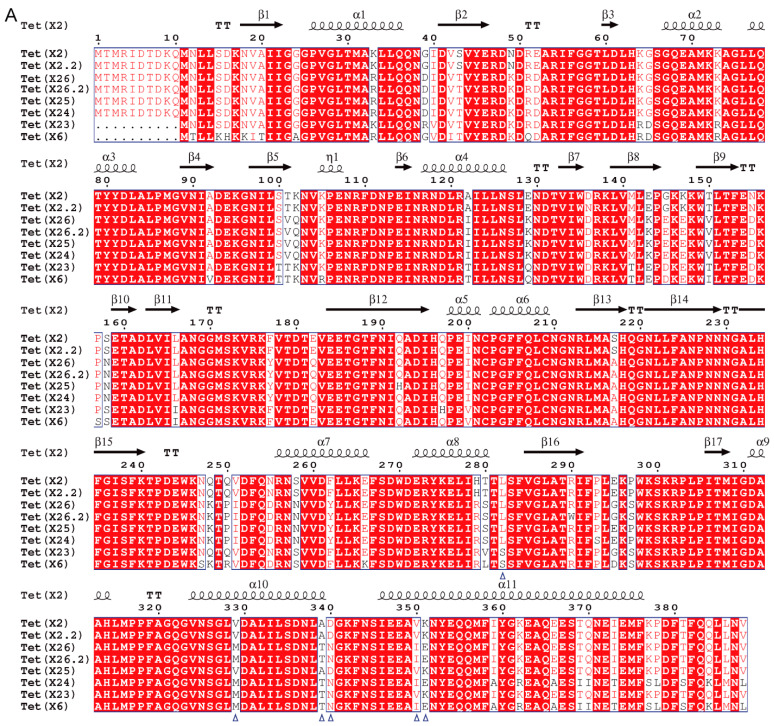

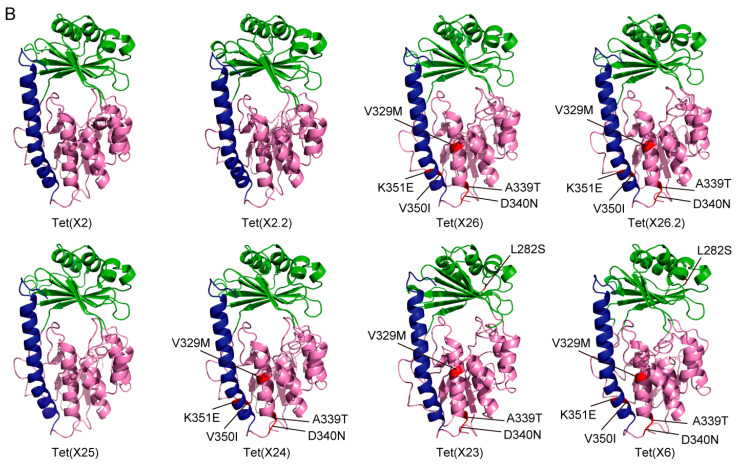
Structural characteristics of Tet(X) variants. (**A**) Multiple sequence alignment of Tet(X) variants. Secondary structure elements of Tet(X2) (2XDO) are present on top, with triangles indicating the reported key amino acid sites. Identical residues are boxed in red. Similar residues in a group or across groups are marked with red characters and blue frames, respectively; (**B**) homology modelling of Tet(X) variants with Tet(X2) (4A6N). The substrate-binding domain (green), FAD-binding domain (pink), and C-terminal helix (blue) are marked, and the reported key amino acid sites are also displayed.

There were eight *tet*(X) variants identified in 37 *tet*(X)-positive *Empedobacter* sp. isolates, including *tet*(X2) (*n* = 14), *tet*(X6) (*n* = 2), and six novel variants (Figure 1). The results of multiple sequence alignment revealed that the novel *tet*(X) variants shared 66.5–99.7% amino acid sequence identities with previously reported ones (Figure S3; Table S3). According to the assignment rule of gene numbers, they were designated as *tet*(X2.2) (*n* = 1), *tet*(X23) (*n* = 1), *tet*(X24) (*n* = 1), *tet*(X25) (*n* = 3), *tet*(X26) (*n* = 22), and *tet*(X26.2) (*n* = 2), respectively (Figure 1). Homology modeling of their encoding proteins with Tet(X2) illustrated an analogous architecture, consisting of the substrate-binding domain, FAD-binding domain, and C-terminal α-helix (Figure 2). Except for Tet(X2.2) and Tet(X25), they shared similar amino acid substitutions (e.g., L282S, V329M, A339T, D340N, V350I, or K351E) as previously reported [44,48]. Functionally, the MICs of tigecycline against the *tet*(X23), *tet*(X24), *tet*(X25), *tet*(X26), and *tet*(X26.2) constructs increased by 64-fold when compared with that of the negative control *E. coli* JM109 carrying an empty pBAD24 vector (Table 1). In addition, the constructs exhibited a 32–128-fold increase for tetracycline, doxycycline, and minocycline, while the *tet*(X2.2) construct was only 2–8-fold (Table 1). By querying the NCBI database, *tet*(X2.3)-positive *E. falsenii* (*n* = 2), *tet*(X14)-positive *E. stercoris* (*n* = 2) as well as *tet*(X2)-positive *E. falsenii* (*n* = 5), *E. stercoris* (*n* = 5), and Taxon 1 (*n* = 2) were also detected, including one *E. stercoris* strain co-harboring *tet*(X2) and *tet*(X14) (Figure 1).

Besides, *bla*_EBR-4_ (*n* = 3) and six novel variants were randomly distributed in all 37 *tet*(X)-positive *Empedobacter* sp. strains. As per the rule mentioned above, the novel *bla*_EBR_ variants were designated as *bla*_EBR-5.2_ (*n* = 6), *bla*_EBR-5.3_ (*n* = 7), *bla*_EBR-6_ (*n* = 3), *bla*_EBR-7_ (*n* = 1), *bla*_EBR-7.2_ (*n* = 7), and *bla*_EBR-7.3_ (*n* = 10), respectively, which shared > 81.6% amino acid sequence identities and three-dimensional structures with previously reported variants (Figure S4). The MIC results revealed that the *bla*_EBR-5.2_, *bla*_EBR-6_, and *bla*_EBR-7.3_ variants exhibited low-level activities for meropenem (2–4-fold increase) and cefotaxime (≥4-fold increase), but the *bla*_EBR-5.3_, *bla*_EBR-7_, and *bla*_EBR-7.2_ constructs exhibited no MIC changes (Table 1). Furthermore, the *Empedobacter* sp. strain carrying *bla*_EBR-3_ (*n* = 2), *bla*_EBR-3.2_ (*n* = 1), *bla*_EBR-3.3_ (*n* = 2), *bla*_EBR-3.4_ (*n* = 1), *bla*_EBR-4_ (*n* = 1), *bla*_EBR-5_ (*n* = 1), *bla*_EBR-6.2_ (*n* = 1), *bla*_EBR-7.4_ (*n* = 2), *bla*_EBR-7.5_ (*n* = 2), *bla*_EBR-8_ (*n* = 1), or *bla*_EBR-9_ (*n* = 1) was available in the NCBI database (Figure 1).

It is noted that 32 out of 37 *tet*(X)- and *bla*_EBR_-positive *Empedobacter* sp. strains carried seven novel *estT* variants, including *estT-1.2* (*n* = 26), *estT-1.3* (*n* = 1), *estT-1.4* (*n* = 1), *estT-2* (*n* = 2), *estT-3* (*n* = 2), *estT-4* (*n* = 1), and *estT-5* (*n* = 1, Figure 1). Although they shared >82.2% amino acid sequence identities with the first reported *estT-1* (CP094932), *estT-1.2*, *estT-1.4*, and *estT-2* lacked an N-terminal random coil (Figure S5). The MICs indicated all exhibited low-level activities for 16-atom-containing tylosin (≥2-fold increase), tilmicosin (2–16-fold increase), and tildipirosin (4–16-fold increase, Table 1). Sporadically, *E. falsenii* strains carrying *estT-1.2* (*n* = 2) and *E. stercoris* strains carrying *estT-1.3* (*n* = 1), *estT-1.6-* (*n* = 1), both *estT-1.2* and *estT-1.7* (*n* = 1) were queried online (Figure 1).

### 3.3. Diverse Phylogeny of tet(X)-Positive Empedobacter sp. Strains

To explore the bacterial relationship between *tet*(X)-positive *Empedobacter* sp. strains, a WGS-based SNP analysis of 37 *E. stercoris*, 8 Taxon 1, and 7 *E. falsenii* from this study (*n* = 37) and the NCBI database (*n* = 15) was conducted. The phylogenetic tree revealed that *E. stercoris*, Taxon 1, and *E. falsenii* formed three separate clusters, with the maximal 21,822 SNPs (Figure 1). In part, eight pairs of strains exhibited a high similarity (SNPs ≤ 2), respectively, such as *E. stercoris* B32-4 and C25-4; *E. stercoris* C10-2 and C16-3; *E. stercoris* ES182 and ES183; *E. stercoris* YWS5-3 and YWS11-1; *E. falsenii* 1681-1 and EF1; *E. falsenii* N76-4 and N85-1; Taxon 1 LDH16-3 and LDH24-2; Taxon 1 B1-4 and B10-4. Significantly, there existed a potential evolutionary branch of Taxon 1 strains (SNPs ≤ 6694), which included *tet*(X2)-positive B4b2P8686 and Q1655 from humans, *tet*(X2)-positive C8-4 from chickens, and chicken-derived B1-4 and B10-4 co-harboring *tet*(X2) and *tet*(X6).

### 3.4. Genetic Characteristics of tet(X), bla_EBR_, and estT Genes

**Figure 3:**
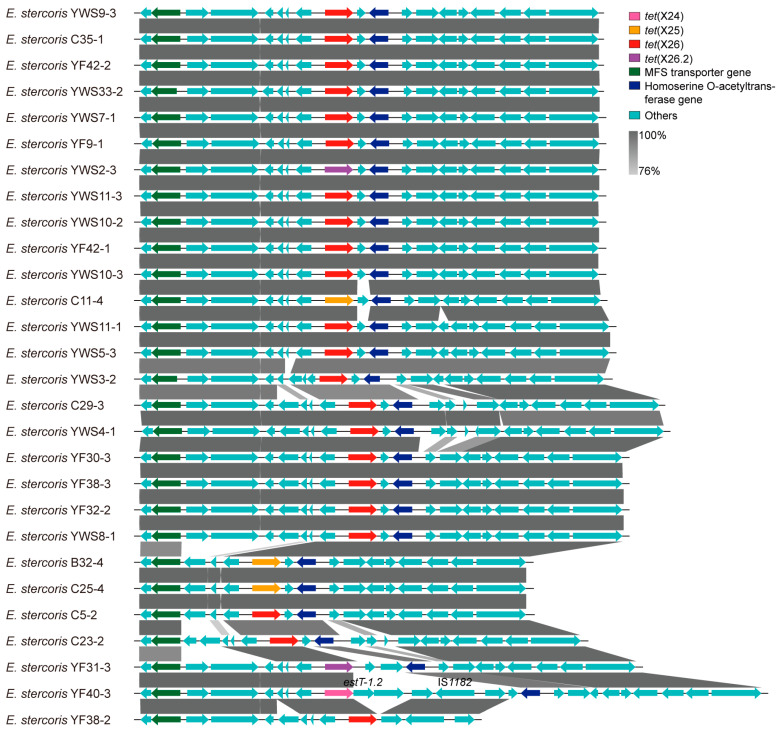
Conserved genetic backgrounds of *tet*(X24), *tet*(X25), *tet*(X26), and *tet*(X26.2) genes in *Empedobacter* sp. isolates. Regions of >76% nucleotide identity are marked by shading.

Although the resistance gene variants and bacterial phylogenetic relationship were diverse, the genetic environments of *tet*(X) and *bla*_EBR_ were more conserved than *estT*. Briefly, all *tet*(X24), *tet*(X25), *tet*(X26), and *tet*(X26.2) genes shared a similar environment inserted between the MFS transporter gene and the homoserine O-acetyltransferase gene (e.g., *E. stercoris* YWS9-3; Figure 3). The *bla*_EBR-4_, *bla*_EBR-5.2_, *bla*_EBR-5.3_, *bla*_EBR-6_, *bla*_EBR-7_, *bla*_EBR-7.2_, and *bla*_EBR-7.3_ genes were also conserved between the hypothetical gene I and the agmatine deiminase gene (e.g., *E. stercoris* B32-4; Figure S6). By contrast, the genetic environments of *estT* genes were various and only *estT-1.2* and *estT-3* (e.g., *E. stercoris* YWS11-3) were similar to *estT-1,* which is adjacent to *floR* from environmental *Sphingobacterium faecium* WB1 (GCA_025340125.1; Figure S7).

**Figure 4:**
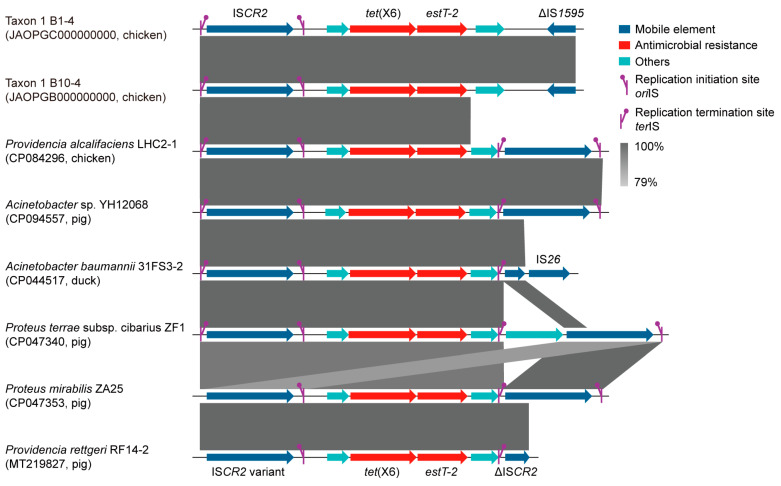
IS*CR2*-related transposition events across different bacterial species. Regions of >79% nucleotide identity are marked by shading. The Δ symbol indicates that the gene is truncated.

According to the insertion sequences analyzed by ISfinder, an IS*CR2*-mediated transposon unit [namely IS*CR2*-hp-*tet*(X6)-*estT-2*-hp-ΔIS*1595*] was identified in chicken-derived Taxon 1 strains B1-4 and B10-4 (Figure 4). A series of *Acinetobacter* sp. (e.g., CP094557), *Proteus* sp. (e.g., CP047340), and *Providencia* sp. (e.g., CP084296) genomes carrying the *tet*(X6) transposon unit from food-producing animals were further identified in the NCBI database (Figure 4). Additionally, one IS*1182*-mediated transposon unit of *tet*(X26) and *estT-1.2* genes were found in chicken-derived *E. stercoris* YF40-3 (Figure 3). The results of our conjugation experiments revealed that none of *tet*(X) genes could be transferred into the recipient *A. baylyi* ADP1 and *E. coli* C600. Nanopore sequencing of environmental *E. stercoris* YWS9-3 confirmed that *tet*(X2) and *tet*(X26) were distantly located on the chromosome cYWS9-3 (CP106831), which may lead to failure.

## 4. Discussion

The *Empedobacter* genus appears as a group of sporadically reported pathogens, such as *E. stercoris*, *E. falsenii*, *E. brevis*, *E. sedimenti*, and *E. tilapiae*. Since the emergence of *tet*(X2) and *tet*(X3.2) in *E. brevis*, *tet*(X2) and its N-truncated variant have been detected in *E. falsenii* as well as *tet*(X2) and *tet*(X14) in *E. stercoris* [17,21,22,23]. Except for *tet*(X3.2) (8 mg/L), the MICs of tigecycline against the *E. coli* clones with *tet*(X14) (2 mg/L), *tet*(X2) (0.25 mg/L), or its variant (1 mg/L) failed to reach the USFDA’s resistance breakpoint of 8 mg/L. Worrisomely, our study demonstrated a higher prevalence of *tet*(X)-mediated tigecycline-resistant *E. stercoris* and Taxon 1 strains in chickens and adjacent environments (especially sewage), indicating a potential transmission risk. As a major province of distribution, Shandong urgently needs to detect them. What is more, the *tet*(X)-positive *E. stercoris* and Taxon 1 strains were not detected in chicken carcasses obtained after slaughter, revealing a key process to prevent bacterial contamination.

To date, a total of 52 *tet*(X) variants have been reported from different bacterial hosts (Table S3, collected on 16 April 2024), of which 44 non-duplicate and non-frameshifted variants were confirmed (Figure S3). In this study, there were eight *tet*(X) variants identified, of which six were novel, and *tet*(X23), *tet*(X24), *tet*(X25), *tet*(X26), and *tet*(X26.2) were able to confer tigecycline resistance (8 mg/L). A series of key amino residue mutations such as L282S, V329M, A339T, D340N, V350I, and K351E have been reported as leading to enhanced Tet(X2) activity [44,48]. Homology modeling of Tet(X6), Tet(X23), Tet(X24), Tet(X26), and Tet(X26.2) with the template Tet(X2) using the online server SWISS-MODEL identified similar substitutions, but none were for Tet(X2.2) and Tet(X25) and requires further study (Figure 2B).

Moreover, there were 19 *bla*_EBR_ variants as shown in Figure S4A, including five variants that have been previously reported in *E. brevis* (*bla*_EBR-1_), *E. falsenii* (*bla*_EBR-2_, *bla*_EBR-3_, *bla*_EBR-4_), and *E. stercoris* (*bla*_EBR-5_) [16,18,19,20]. We also reported six novel *bla*_EBR_ variants in *E. stercoris* and Taxon 1, of which *bla*_EBR-5.2_, *bla*_EBR-6_, and *bla*_EBR-7.3_ exhibited 2–4-fold higher MICs for one of the last-resort antibiotic meropenem than *bla*_EBR-1_ but 2–4-fold lower than *bla*_EBR-4_ (Table 1) [20]. Apart from the *tet*(X) and *bla*_EBR_ genes, a total of 11 *estT* variants were collected (Figure S5A), including seven novel variants in this study capable of inactivating 16-atom-containing macrolides like the first reported *estT* [24]. One another *estT* variant (designated *estT-1.5* by us) was identified on the chromosome of *Pasteurella multocida* 17BRD-035 (CP082272), but its hydrolyzation activity needs to be confirmed [49]. The available data indicate that *Empedobacter* sp. strains appear as a reservoir of *tet*(X), *bla*_EBR_, and *estT* genes.

It is noted that the genetic environments of *tet*(X) and *bla*_EBR_ variants were more conservative than *estT* variants on *Empedobacter* sp. genomes. However, their evolutionary routes remain to be explored. Our previous studies reported the IS*CR2*-mediated transposition events of *tet*(X3), *tet*(X4), and *tet*(X5), which probably originated from the *Weeksellaceae* (formerly *Flavobacteriaceae*) genomes but lack direct evidence [6,11,50]. Herein, we identified an IS*CR2*-mediated transposon unit in Taxon 1 B1-4 and B10-4 belonging to the *Weeksellaceae* family and supporting the evolutionary hypothesis. Although failing to be transferred by conjugation, a similar transposition structure was detected by querying in the NCBI database in *Acinetobacter* sp., *Providencia* sp., and *Proteus* sp. bacteria of chicken, pig, and duck origin, indicating a risk of cross-species dissemination in food-producing animals (Figure 4).

## 5. Conclusions

Taken together, our study demonstrates a diversity of *tet*(X)-, *bla*_EBR_-, and *estT*-positive MDR *Empedobacter* sp. strains in chickens and surrounding environments (especially sewage), and the slaughter process in slaughterhouses may be an effective means to clear the contamination. Particularly, there were eight *tet*(X) variants together with seven *bla*_EBR_ variants and seven *estT* variants identified in this study, and the genetic environments of *tet*(X) and *bla*_EBR_ are more conserved than *estT*. Given the emergence of IS*CR2*-mediated *tet*(X6) in the novel bacterial species Taxon 1, future efforts are needed to improve the surveillance of *Empedobacter* sp. strains from all related sectors and to evaluate their clinical impact.

## Figures and Tables

**Table 1:** MICs of *E. coli* JM109 + pBAD24 and its clones.

Clone	MIC (mg/L) ^†^								
	TC	DOX	MIN	TGC	MEM	CTX	TYL	TIL	TIP
Empty	2	0.5	0.25	0.125	0.25	≤0.125	128	8	0.25
*tet*(X2)	16	4	0.5	0.25	-	-	-	-	-
*tet*(X2.2)	16	4	0.5	0.25	-	-	-	-	-
*tet*(X6)	64	16	16	8	-	-	-	-	-
*tet*(X23)	64	16	16	8	-	-	-	-	-
*tet*(X24)	128	32	32	8	-	-	-	-	-
*tet*(X25)	128	32	16	8	-	-	-	-	-
*tet*(X26)	128	32	16	8	-	-	-	-	-
*tet*(X26.2)	128	32	32	8	-	-	-	-	-
*bla*_EBR-4_	-	-	-	-	2	2	-	-	-
*bla*_EBR-5.2_	-	-	-	-	0.5	1	-	-	-
*bla*_EBR-5.3_	-	-	-	-	0.25	≤0.125	-	-	-
*bla*_EBR-6_	-	-	-	-	1	0.5	-	-	-
*bla*_EBR-7_	-	-	-	-	0.25	≤0.125	-	-	-
*bla*_EBR-7.2_	-	-	-	-	0.25	≤0.125	-	-	-
*bla*_EBR-7.3_	-	-	-	-	0.5	1	-	-	-
*estT-1.2*	-	-	-	-	-	-	512	64	1
*estT-1.3*	-	-	-	-	-	-	>512	32	1
*estT-1.4*	-	-	-	-	-	-	512	16	1
*estT-2*	-	-	-	-	-	-	256	64	1
*estT-3*	-	-	-	-	-	-	>512	128	4
*estT-4*	-	-	-	-	-	-	>512	128	4
*estT-5*	-	-	-	-	-	-	>512	32	1

^†^ TC, tetracycline; DOX, doxycycline; MIN, minocycline; TGC, tigecycline; MEM, meropenem; CTX, cefotaxime; TYL, tylosin; TIL, tilmicosin; TIP, tildipirosin.

## Data Availability

The original contributions presented in the study are included in the article/Supplementary Materials; further inquiries can be directed to the corresponding author.

## Supplementary Materials

The following supporting information can be downloaded at: https://www.mdpi.com/article/10.3390/foods13193201/s1. Figure S1: Resistance rates of ten antibiotics against 37 *tet*(X)-positive *Empedobacter* sp. strains; Figure S2: MICs of tylosin (A), tilmicosin (B), and tildipirosin (C) against 37 *tet*(X)-positive *Empedobacter* sp. strains; Figure S3: Maximum-likelihood phylogenetic tree of the Tet(X) variants; Figure S4: Structural characteristics of the EBR proteins; Figure S5: Structural characteristics of the EstT proteins; Figure S6: Conserved genetic backgrounds of the *bla*_EBR_ genes; Figure S7: Diverse genetic backgrounds of *estT* genes; Table S1: Distribution of *tet*(X)-positive *Empedobacter* sp. isolates of different sources; Table S2: Primers used in this study; Table S3: Summary of the *tet*(X) variants (*n* = 52, accessed on 16 April 2024). Refs. [4,7,9,11,17,21,23,51,52,53,54,55,56,57,58,59,60,61,62,63] are cited in Supplementary Materials.
