# Supplementary material for: Concurrence of Inactivation Enzyme-Encoding Genes tet(X), blaEBR, and estT in Empedobacter Species from Chickens and Surrounding Environments
Source: Foods. 2024 Oct 9;13(19):3201. doi: 10.3390/foods13193201 (PMC11475475; doi:10.3390/foods13193201)
Supplement: Supplementary file 1 [file foods-13-03201-s001.zip › foods-3185738-supplementary.pdf]

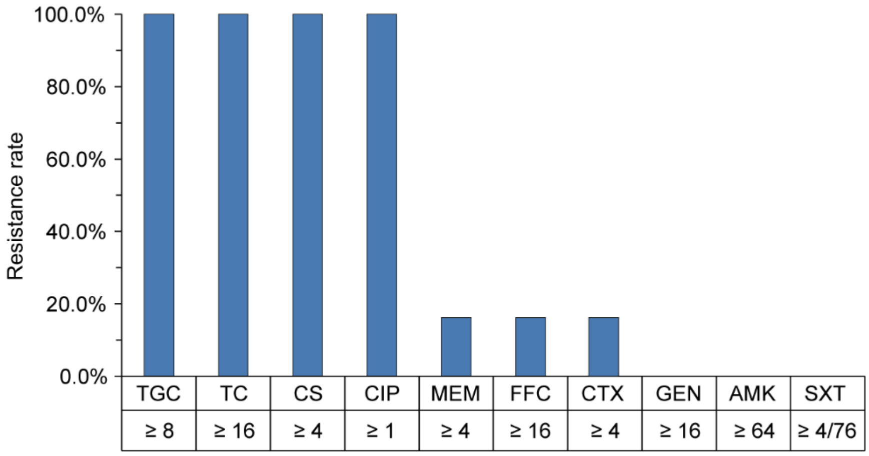

Figure S1. Resistance rates of ten antibiotics against 37 *tet*(X)-positive *Empedobacter* sp. strains. The resistance breakpoints of different antibiotics are also present. TGC, tigecycline; TC, tetracycline; CS, colistin; CIP, ciprofloxacin; MEM, meropenem; FFC, florfenicol; CTX, cefotaxime; GEN, gentamicin; AMK, amikacin; SXT, trimethoprim/sulfamethoxazole.

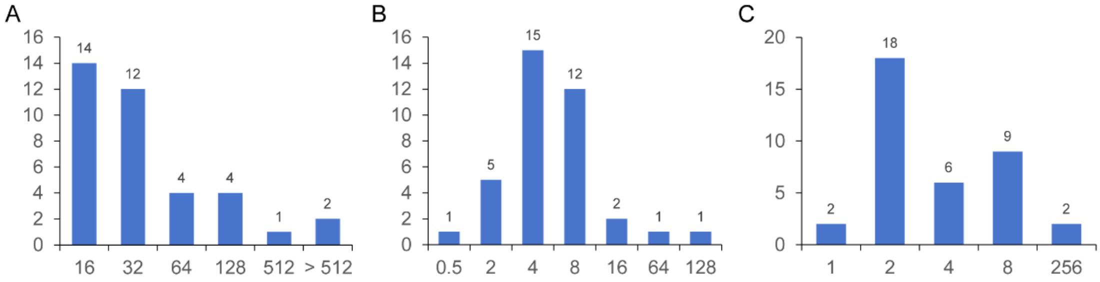

Figure S2. MICs of tylosin (A), tilmicosin (B), and tildipirosin (C) against 37 *tet*(X)-positive *Empedobacter* sp. strains. Bacterial numbers per dilution (mg/L) are present.

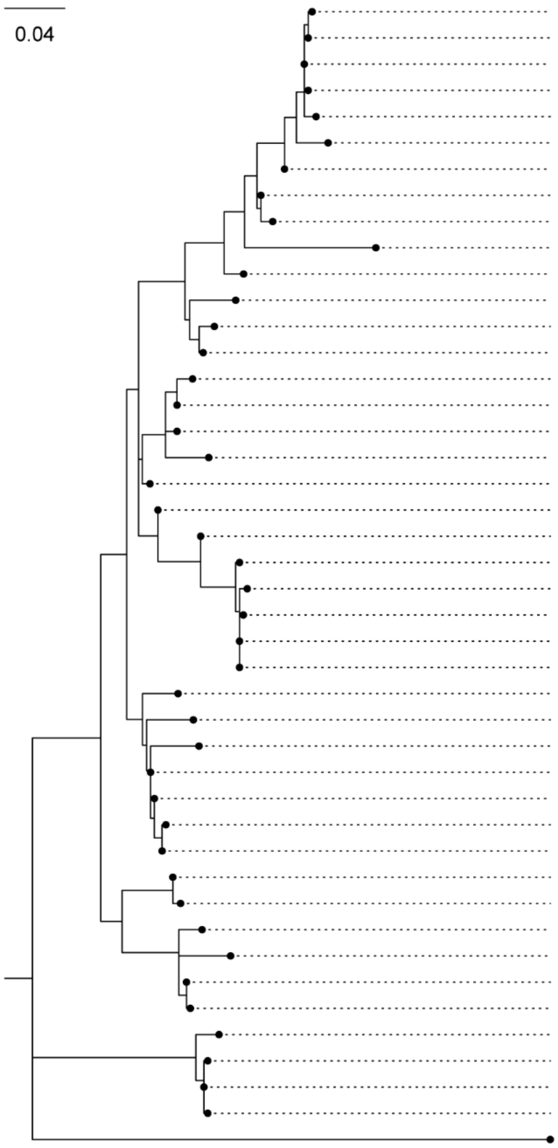

Variant	Accession number
Tet(X6.2)	CP081928
Tet(X6.3)	CP081929
Tet(X6)/Tet(X5.2)	MN507533/CP048670
Tet(X13)	KU547125
Tet(X) variant	AP025181
Tet(X5.3)/Tet(X6) variant	CP048661/CP048828
Tet(X) variant	CP085032
Tet(X7)	KU547176
Tet(X9)	MT292309
Tet(X22)	CP114976
Tet(X8)	KU548516
Tet(X15)	CP060811
Tet(X5)	CP040912
Tet(X5.4)	CP094261
Tet(X12)	KU548377
Tet(X12.2)	CP072185
Tet(X20)	JAGFUR010000001
Tet(X4)	MK134376
Tet(X19)	CP081923
Tet(X11)	MT292307
Tet(X21)	CP072188
Tet(X10.2)	CP081927
Tet(X0)	M37699
Tet(X2.2)	JAOPGU000000000
Tet(X2)/Tet(X10)	AJ311171/KU548536
Tet(X) variant	CP040909
Tet(X23)	JAOPGT000000000
Tet(X16)	JAGFUM010000001
Tet(X18)	CP072192
Tet(X14)	CP053698
Tet(X14.2)	CP007503
Tet(X14.3)	CP081929
Tet(X14.4)	JAGFUF010000016
Tet(X17)	JAGFUH010000017
Tet(X17.2)	CP081928
Tet(X25)	JAOPGI000000000
Tet(X24)	JAOPGW000000000
Tet(X26)	CP106831
Tet(X26.2)	JAOPGR000000000
Tet(X3.2)	CP043635
Tet(X3.9)	CP094838
Tet(X3)	MK134375
Tet(X3.7)	CP094838
Tet(X1)	AJ311171

Figure S3. Maximum-likelihood phylogenetic tree of the Tet(X) variants. GenBank accession numbers are provided, of which the novel Tet(X) variants (n = 6) are marked in red. The proteins encoded by previously reported *tet*(X3.3) (CP094246), *tet*(X3.4) (CP094246), *tet*(X3.5) (CP094246), *tet*(X3.6) (CP094246), and *tet*(X3.8) (CP094838) are frameshifted, and therefore aren't involved in the phylogenetic analysis. Bar, 0.04 amino acid substitutions per site.

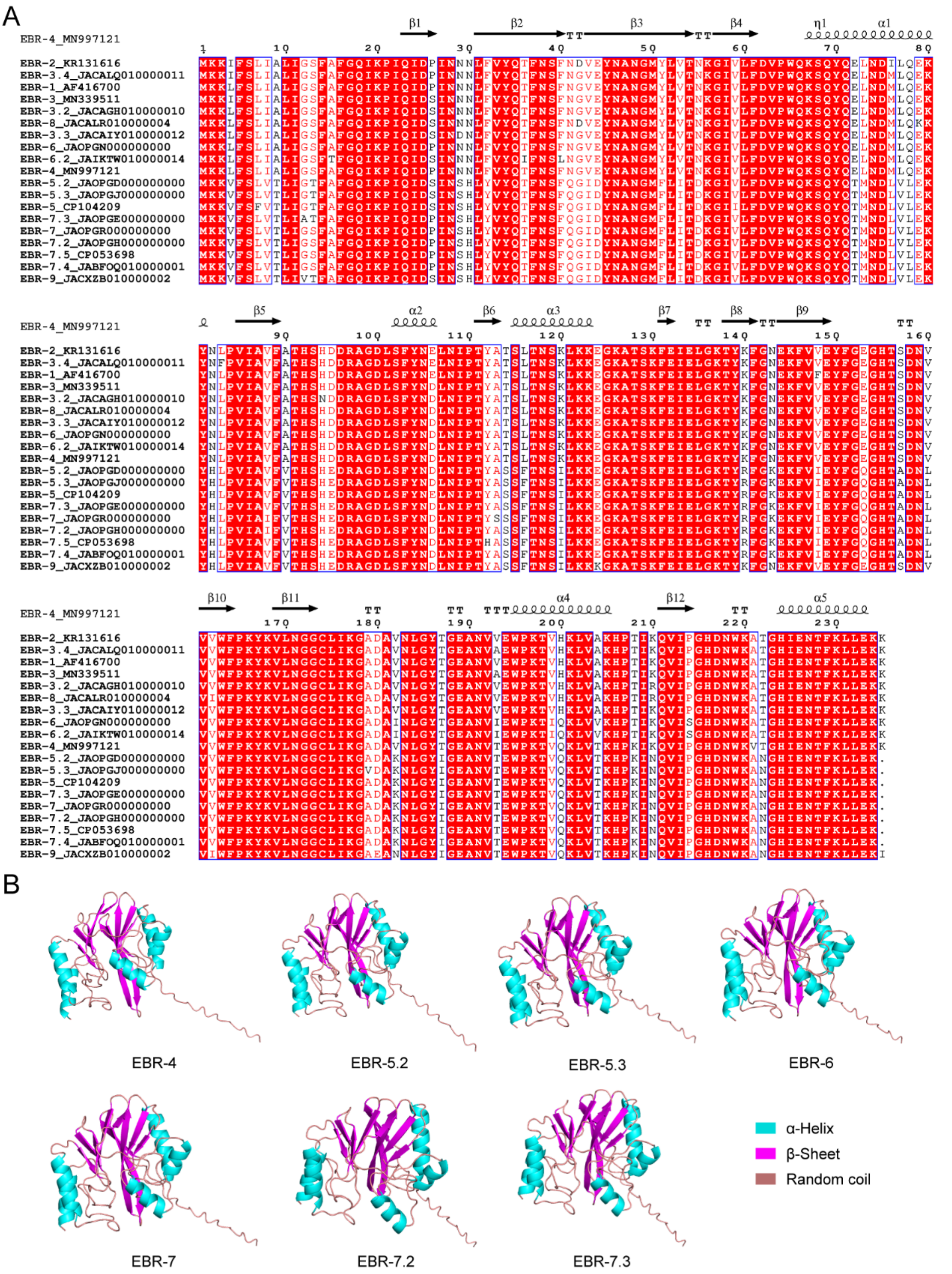

Figure S4. Structural characteristics of the EBR proteins. (A) Result of multiple **s**equence alignment by ESPript version 3.0. GenBank accession numbers of the EBR proteins are provided. (B) Prediction of three-dimensional structures by AlphaFold 3. The newly identified EBR variants are colored by their secondary structures and EBR-4 (MN997121) is used as the reference protein.

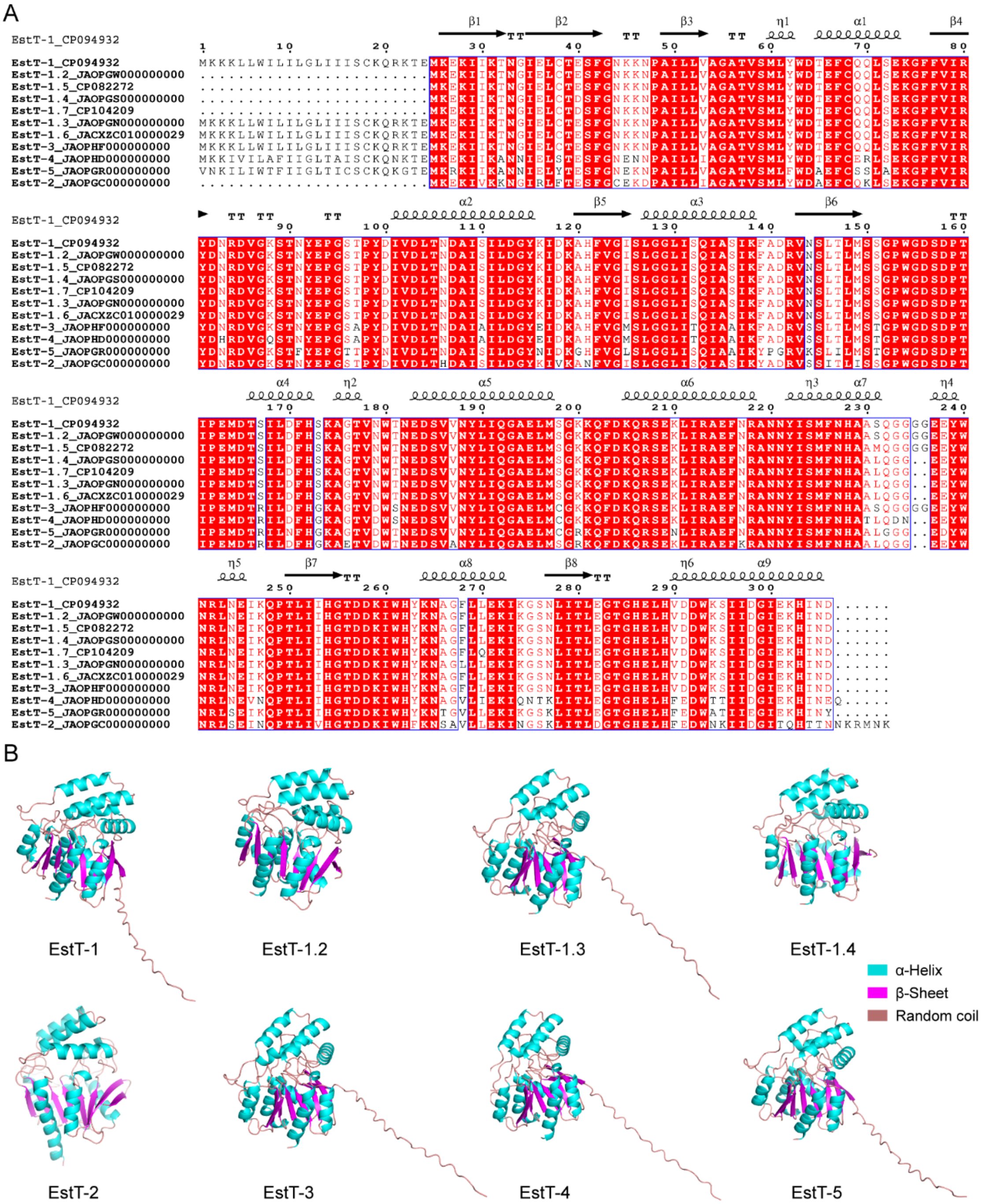

Figure S5. Structural characteristics of the EstT proteins. (A) Multiple **s**equence alignment of the EstT proteins by ESPript version 3.0. GenBank accession numbers of them are provided. (B) Prediction of three-dimensional structures by AlphaFold 3. The newly identified EstT variants are colored by their secondary structures and EstT-1 (CP094932) is used as the reference protein.

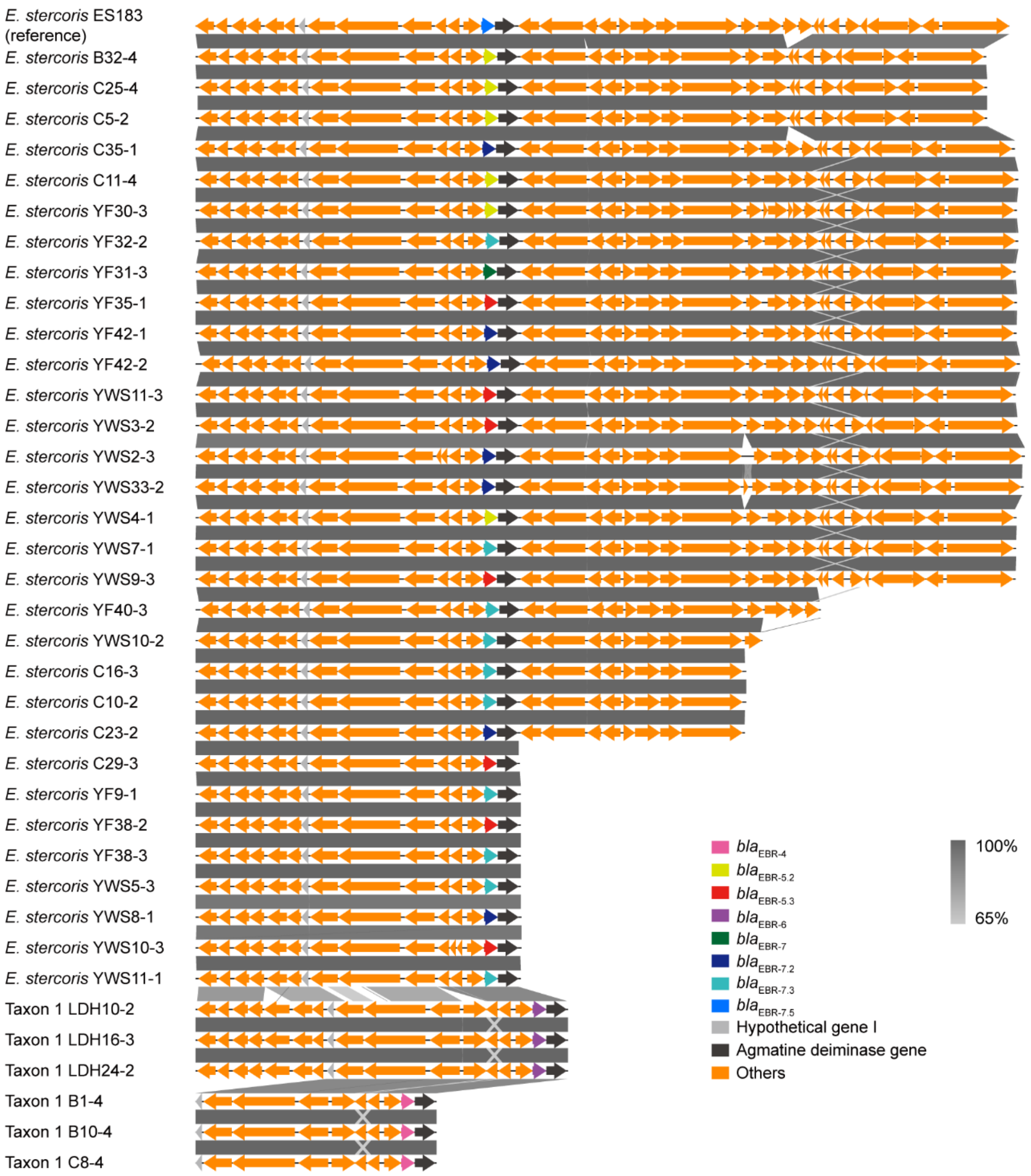

Figure S6. Conserved genetic backgrounds of the *bla*EBR genes. The *bla*EBR-7.5-positive *E. stercoris* ES183 (GCA_014621655.1) is used as the reference strain. Regions of > 65% nucleotide identity are marked by shading.

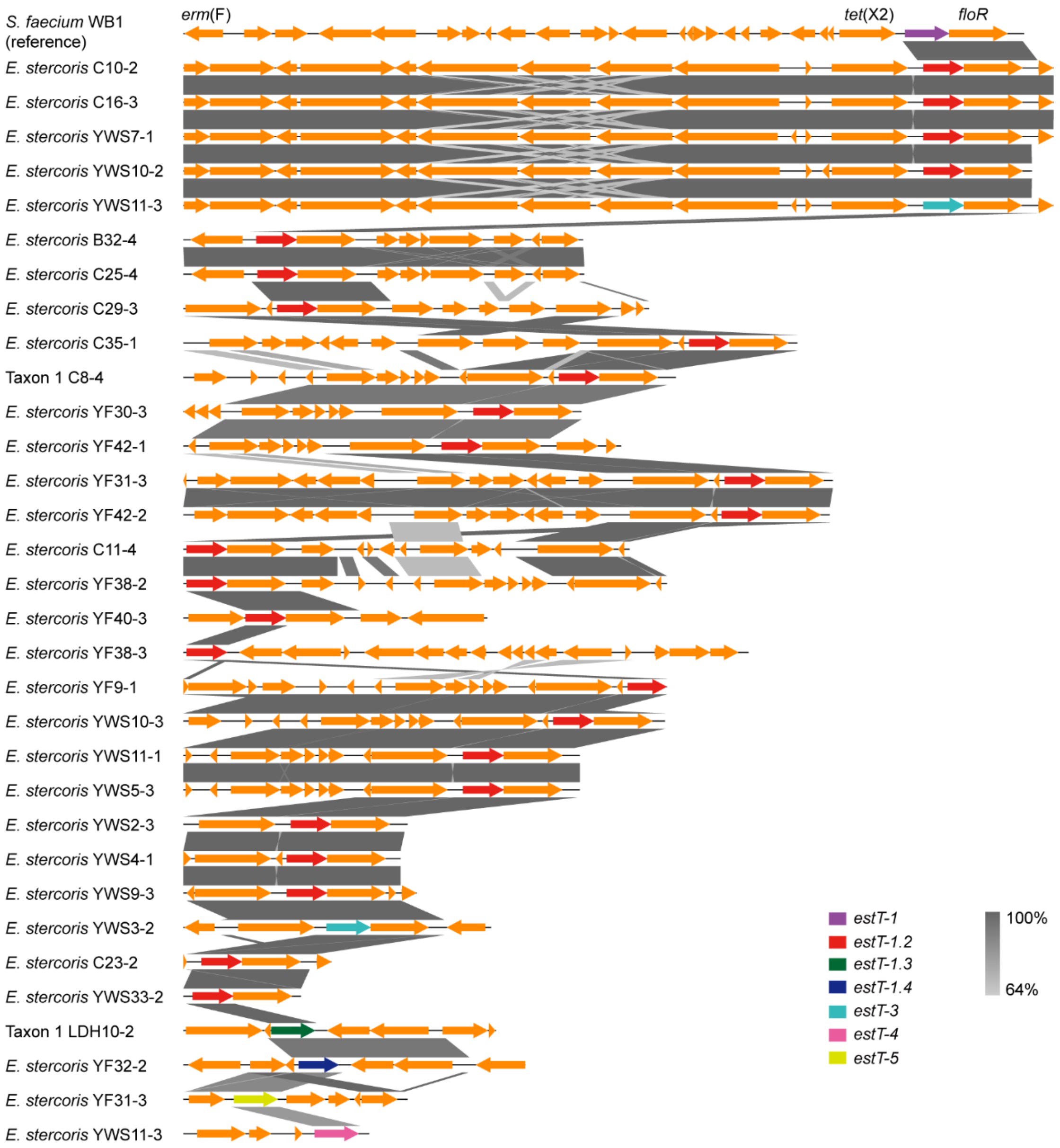

Figure S7. Diverse genetic backgrounds of *estT* genes. The *estT-1*-positive *S. faecium* WB1 (GCA_025340125.1) is used as the reference strain. Regions of > 64% nucleotide identity are marked by shading.

Table S1. Distribution of *tet*(X)- positive *Empedobacter* sp. isolates of different sources.

**Province**	**Detection rate (percentage of positive strain/sample)**				
	**Chicken feces**	**Chicken carcass**	**Sheep feces**	**Soil**	**Sewage**
Shandong	11.3% (22/195)	0.0% (0/60)	0.0% (0/0)	5.6% (1/18)	61.1% (11/18)
Jiangsu	2.5% (3/122)	0.0% (0/0)	0.0% (0/0)	0.0% (0/30)	0.0% (0/18)
Ningxia	0.0% (0/0)	0.0% (0/0)	0.0% (0/95)	0.0% (0/0)	0.0% (0/0)
In total	7.9% (25/317)	0.0% (0/60)	0.0% (0/95)	2.1% (1/48)	30.6% (11/36)

Table S2. Primers used in this study.

Primer	Nucleotide sequence (5′ to 3′) †	Size (bp)
tet(X)-uniF	TTCAGGTCAGGAAGCAATGAAAA	780 [1]
tet(X)-uniR	TTTACGCCTTGTCCTGCAAAAGG	
16S rDNA-F	AGAGTTTGATCCTGGCTCAG	1450 [2]
16S rDNA-R	GGTTACCTTGTTACGACTT	
tet(X2.2)-F	ttgggctagcaggaggaattcATGACAATGCGAATAGATACAGACAA	1167 ††
tet(X2.2)-R	cttgcatgcctgcaggtcgacTTATACATTTAACAATTGCTGAAACGTAA	
tet(X23)-F	ttgggctagcaggaggaattcATGAATTTACTTAGTGATAAGAACGTTGC	1137
tet(X23)-R	cttgcatgcctgcaggtcgacTTATACATTTAACAATTGCTGAAACGTAA	
tet(X24)-F	ttgggctagcaggaggaattcATGACAATGCGAATAGATACAGACAA	1167
tet(X24)-R	cttgcatgcctgcaggtcgacTTATAGATTCATTAGTTTTGGAACGAA	
bla$_{EBR-4}$-F	ttgggctagcaggaggaattcATGAAGAAATTATTTTCACTTATAGCATTG	708 †††
bla$_{EBR-4}$-R	cttgcatgcctgcaggtcgacTTATTTCTTTTCTAAAAGTTTAAACGTATTTT	
bla$_{EBR-5.2}$-F	ttgggctagcaggaggaattcATGAAAAAAGTATTTTCTCTTGTCACATT	705 ††††
bla$_{EBR-5.2}$-R	cttgcatgcctgcaggtcgacTTATTTTTCTAAAAGTTTAAACGTATTTTCTAT	
estT-1.2-F	ttgggctagcaggaggaattcATGAAAGAGAAAATAATTAAAACAAACGG	849
estT-1.2-R	cttgcatgcctgcaggtcgacTCAGTCATTTATGTGTTTTTCTATTCCA	
estT-1.3-F	tacccgtttttttgggctagcATGAAAAAAAAACTACTTTGGATATTAATTT	915 †††††
estT-1.3-R	cttgcatgcctgcaggtcgacTCAGTCATTTATGTGTTTTTCTATTCCA	
estT-1.4-F	tacccgtttttttgggctagcATGAAAGAGAAAATAATTAAAACAAACGG	843
estT-1.4-R	cttgcatgcctgcaggtcgacTCAGTCATTTATGTGTTTTTCTATTCCA	
estT-2-F	tacccgtttttttgggctagcATGAAAGAGAAAATAGTTAAAAAAAACGG	861
estT-2-R	cttgcatgcctgcaggtcgacCTATTTGTTCATTCTTTTGTTATTTGTTG	
estT-4-F	tacccgtttttttgggctagcATGAAGAAAATAGTAATTTTAGCATTTATAATT	918
estT-4-R	cttgcatgcctgcaggtcgacTTATTGCTCATTTATATGTTTTTCAATTC	
estT-5-F	tacccgtttttttgggctagcGTGAATAAAATACTAATTTGGACTTTTATAATT	915
estT-5-R	cttgcatgcctgcaggtcgacTTAGTAATTTATGTGCTTTTCAATTCCTT	
pBAD24-F	CGGCGTCACACTTTGCTATG	Cloning confirmation [3]
pBAD24-R	ACGGCGTTTCACTTCTGAGT	

† The homologous sequences are underlined.

†† The primers are used for cloning of *tet*(X2.2), *tet*(X25), *tet*(X26), and *tet*(X26.2) genes.

††† The primers are used for cloning of *bla*$_{EBR-4}$ and *bla*$_{EBR-6}$ genes.

†††† The primers are used for cloning of *bla*$_{EBR-5.2}$, *bla*$_{EBR-5.3}$, *bla*$_{EBR-7}$, *bla*$_{EBR-7.2}$, and *bla*$_{EBR-7.3}$ genes.

††††† The primers are used for cloning of *estT-1.3* and *estT-3* genes.

Table S3. Summary of the *tet*(X) variants (n = 52, accessed on 16 Apr 2024).

Subtype	Accession number	Bacterial species	Size (bp)	Reference
tet(X0)	M37699	*Bacteroides fragilis*	1167	[4]
tet(X1)	AJ311171	*Bacteroides thetaiotaomicron*	1080	[5]
tet(X2) †	AJ311171	*Bacteroides thetaiotaomicron*	1167	[5]
tet(X2.2)	JAOPGU000000000	*Empedobacter falsenii*	1167	This study
tet(X) variant	CP040909	*Empedobacter falsenii*	1137	[6]
tet(X3)	MK134375	*Acinetobacter baumannii*	1167	[7]
tet(X3.2)	CP043635	*Empedobacter brevis*	1167	[8]
tet(X3.3) ††	CP094246	*Acinetobacter variabilis*	1166	[9]
tet(X3.4) ††	CP094246	*Acinetobacter variabilis*	1164	[9]
tet(X3.5) ††	CP094246	*Acinetobacter variabilis*	1165	[9]
tet(X3.6) ††	CP094246	*Acinetobacter variabilis*	1165	[9]
tet(X3.7)	CP094838	*Acinetobacter schindleri*	1167	[9]
tet(X3.8) ††	CP094838	*Acinetobacter schindleri*	1165	[9]
tet(X3.9)	CP094838	*Acinetobacter schindleri*	1167	[9]
tet(X4)	MK134376	*Escherichia coli*	1158	[7]
tet(X5)	CP040912	*Acinetobacter baumannii*	1167	[10]
tet(X5.2) †††	CP048670	*Acinetobacter* sp.	1137	[3]
tet(X5.3) ††††	CP048661	*Acinetobacter* sp.	1137	[3]
tet(X5.4)	CP094261	*Acinetobacter indicus*	1167	[11]
tet(X6) †††	MN507533	*Proteus* genomospecies 6	1137	[12]
tet(X6.2)	CP081928	*Riemerella anatipestifer*	1137	[13]
tet(X6.3)	CP081929	*Riemerella anatipestifer*	1137	[13]
tet(X6) variant ††††	CP048828	*Acinetobacter baumannii*	1137	[14]
tet(X) variant	AP025181	*Escherichia coli*	1137	[15]
tet(X) variant	CP085032	*Proteus terrae* subsp. cibarius	1137	[16]
tet(X7)	KU547176	Human enteric microorganism	1137	[17]
tet(X8)	KU548516	Human enteric microorganism	1137	[17]
tet(X9)	MT292309	Human enteric microorganism	1137	[17]
tet(X10) †	KU548536	Human enteric microorganism	1167	[17]
tet(X10.2)	CP081927	*Riemerella anatipestifer*	1167	[13]
tet(X11)	MT292307	Human enteric microorganism	1167	[17]
tet(X12)	KU548377	Human enteric microorganism	1167	[17]
tet(X12.2)	CP072185	*Riemerella anatipestifer*	1167	[13]
tet(X13)	KU547125	Human enteric microorganism	1137	[17]
tet(X14)	CP053698	*Empedobacter stercoris*	1167	[18]
tet(X14.2)	CP007503	*Riemerella anatipestifer*	1167	[18]
tet(X14.3)	CP081929	*Riemerella anatipestifer*	1167	[13]
tet(X14.4)	JAGFUF010000016	*Riemerella anatipestifer*	1167	[13]
tet(X15)	CP060811	*Acinetobacter variabilis*	1158	[19]
tet(X16)	JAGFUM010000001	*Riemerella anatipestifer*	1167	[13]
tet(X17)	JAGFUH010000017	*Riemerella anatipestifer*	1167	[13]
tet(X17.2)	CP081928	*Riemerella anatipestifer*	1167	[13]
tet(X18)	CP072192	*Riemerella anatipestifer*	1137	[13]
tet(X19)	CP081923	*Riemerella anatipestifer*	1167	[13]
tet(X20)	JAGFUR010000001	*Riemerella anatipestifer*	1167	[13]
tet(X21)	CP072188	*Riemerella anatipestifer*	1167	[13]
tet(X22)	CP114976	*Pseudomonas caeni*	1137	[20]
tet(X23)	JAOPGT000000000	*Empedobacter falsenii*	1137	This study
tet(X24)	JAOPGW000000000	*Empedobacter falsenii*	1167	This study

tet(X25)	JAOPGI000000000	*Empedobacter falsenii*	1167	This study
tet(X26)	CP106831	*Empedobacter falsenii*	1167	This study
tet(X26.2)	JAOPGR000000000	*Empedobacter falsenii*	1167	This study

[†] *tet*(X2) (AJ311171) and *tet*(X10) (KU548536) share an 100% nucleotide identity.

[††] The variants are frameshifted.

[††] *tet*(X5.2) (CP048670) and *tet*(X6) (MN507533) share an 100% nucleotide identity.

[††††] *tet*(X5.3) (CP048661) and the *tet*(X6) variant (CP048828) share an 100% nucleotide identity.

References

1. Hsieh, Y.C.; Wu, J.W.; Chen, Y.Y.; Quyen, T.L.T.; Liao, W.C.; Li, S.W.; Chen, Y.C.; Pan, Y.J. An Outbreak of *tet*(X6)-Carrying Tigecycline-Resistant *Acinetobacter baumannii* Isolates with a New Capsular Type at a Hospital in Taiwan. *Antibiotics* **2021**, *10*, 1239.
2. Lee, M.J.; Jang, S.J.; Li, X.M.; Park, G.; Kook, J.K.; Kim, M.J.; Chang, Y.H.; Shin, J.H.; Kim, S.H.; Kim, D.M. *et al*. Comparison of *rpoB* gene sequencing, 16S rRNA gene sequencing, *gyrB* multiplex PCR, and the VITEK2 system for identification of *Acinetobacter* clinical isolates. *Diagn. Micr. Infec. Dis.* **2014**, *78*, 29-34.
3. Chen, C.; Cui, C.Y.; Yu, J.J.; He, Q.; Wu, X.T.; He, Y.Z.; Cui, Z.H.; Li, C.; Jia, Q.L.; Shen, X.G. *et al*. Genetic diversity and characteristics of high-level tigecycline resistance Tet(X) in *Acinetobacter* species. *Genome Med.* **2020**, *12*, 111.
4. Speer, B.S.; Bedzyk, L.; Salyers, A.A. Evidence that a novel tetracycline resistance gene found on two *Bacteroides* transposons encodes an NADP-requiring oxidoreductase. *J. Bacteriol.* **1991**, *173*, 176-183.
5. Whittle, G.; Hund, B.D.; Shoemaker, N.B.; Salyers, A.A. Characterization of the 13-kilobase *ermF* region of the *Bacteroides* conjugative transposon CTnDOT. *Appl. Environ. Microb.* **2001**, *67*, 3488-3495.
6. Zeng, Y.; Dong, N.; Zhang, R.; Liu, C.; Sun, Q.; Lu, J.; Shu, L.; Cheng, Q.; Chan, E.W.; Chen, S. Emergence of an *Empedobacter falsenii* strain harbouring a *tet*(X)-variant-bearing novel plasmid conferring resistance to tigecycline. *J. Antimicrob. Chemoth.* **2020**, *75*, 531-536.
7. He, T.; Wang, R.; Liu, D.; Walsh, T.R.; Zhang, R.; Lv, Y.; Ke, Y.; Ji, Q.; Wei, R.; Liu, Z. *et al.* Emergence of plasmid-mediated high-level tigecycline resistance genes in animals and humans. *Nat. Microbiol.* **2019**, *4*, 1450-1456.
8. Li, R.C.; Liu, Z.Y.; Peng, K.; Liu, Y.; Xiao, X.; Wang, Z.Q. Co-occurrence of two *tet*(X) variants in an *Empedobacter brevis* of shrimp origin. *Antimicrob. Agents Ch.* **2019**, *63*, e01636-19.
9. Cheng, Y.; Li, Y.; Yu, R.; Ma, M.;Yang, M.; Si, H. Identification of Novel *tet*(X3) Variants Resistant To Tigecycline in *Acinetobacter* Species. *Microbiol. Spectr.* **2022**, *10*, e0133322.
10. Wang, L.; Liu, D.; Lv, Y.; Cui, L.; Li, Y.; Li, T.; Song, H.; Hao, Y.; Shen, J.; Wang, Y. *et al.* Novel Plasmid-Mediated *tet*(X5) Gene Conferring Resistance to Tigecycline, Eravacycline, and Omadacycline in a Clinical *Acinetobacter baumannii* Isolate. *Antimicrob. Agents Ch.* **2019**, *64*, e01326-19.
11. Cheng, Y.; Li, Y.; Yang, M.; He, Y.; Shi, X.; Zhang, Z.; Zhong, Y.; Zhang, Y.; Si, H. Emergence of novel tigecycline resistance gene *tet*(X5) variant in multidrug-resistant *Acinetobacter indicus* of swine farming environments. *Vet. Microbiol.* **2023**, *284*, 109837.
12. He, D.; Wang, L.; Zhao, S.; Liu, L.; Liu, J.; Hu, G.; Pan, Y. A novel tigecycline resistance gene, *tet*(X6), on an SXT/R391 integrative and conjugative element in a *Proteus* genomospecies 6 isolate of retail meat origin. *J. Antimicrob. Chemoth.* **2020**, *75*, 1159-1164.
13. Li, R.; Jiang, Y.; Peng, K.; Wang, Y.; Wang, M.; Liu, Y.; Wang, Z. Phenotypic and genomic analysis reveals *Riemerella anatipestifer* as the potential reservoir of *tet*(X) variants. *J. Antimicrob. Chemoth.* **2022**, *77*, 374-380.
14. Zheng, X.R.; Zhu, J.H.; Zhang, J.; Cai, P.; Sun, Y.H.; Chang, M.X.; Fang, L.X.; Sun, J.; Jiang, H.X. A novel plasmid-borne *tet*(X6) variant co-existing with *bla*$_{NDM-1}$ and *bla*$_{OXA-58}$ in a chicken *Acinetobacter baumannii* isolate. *J. Antimicrob. Chemoth.* **2020**, *75*, 3397-3399.
15. Usui, M.; Fukuda, A.; Suzuki, Y.; Nakajima, C.; Tamura, Y. Broad-host-range IncW plasmid harbouring *tet*(X) in *Escherichia coli* isolated from pigs in Japan. *J. Glob. Antimicrob. Re.* **2022**, *28*, 97-101.
16. Zheng, X.; Ma, J.; Lu, Y.; Sun, D.; Yang, H.; Xia, F.; Tang, B. Detection of *tet*(X6) variant-producing *Proteus terrae* subsp. cibarius from animal cecum in Zhejiang, China. *J. Glob. Antimicrob. Re.* **2022**, *29*, 124-130.
17. Gasparrini, A.J.; Markley, J.L.; Kumar, H.; Wang, B.; Fang, L.; Irum, S.; Symister, C.T.; Wallace, M.; Burnham, C.D.; Andleeb, S. *et al*. Tetracycline-inactivating enzymes from environmental, human commensal, and pathogenic bacteria cause broad-spectrum tetracycline resistance. *Commun. Biol.* **2020**, *3*, 241.

18. Cheng, Y.; Chen, Y.; Liu, Y.; Guo, Y.; Zhou, Y.; Xiao, T.; Zhang, S.; Xu, H.; Chen, Y.; Shan, T. *et al*. Identification of novel tetracycline resistance gene *tet*(X14) and its co-occurrence with *tet*(X2) in a tigecycline-resistant and colistin-resistant *Empedobacter stercoris*. *Emerg. Microbes Infec.* **2020**, *9*, 1843-1852.
19. Li, R.; Peng, K.; Xiao, X.; Wang, Y.; Wang, Z. Characterization of novel IS*Aba1*-bounded *tet*(X15)-bearing composite transposon Tn*6866* in *Acinetobacter variabilis*. *J. Antimicrob. Chemoth.* **2021**, *76*, 2481-2483.
20. Lu, X.; Zhang, L.; Peng, K.; Wang, Q.; Liu, R.; Wang, Z.; Li, R. Characterisation of a Novel Tigecycline Resistance Gene *tet*(X22) and its Coexistence with $bla_{\text{NDM-1}}$ in a *Pseudomonas caeni* Isolate. *Int. J. Antimicrob. Ag.* **2023**, *62*, 106961.
